# Isolation and Characterization of a *Phapecoctavirus* Infecting Multidrug-Resistant *Acinetobacter baumannii* in A549 Alveolar Epithelial Cells

**DOI:** 10.3390/v14112561

**Published:** 2022-11-19

**Authors:** Phitchayapak Wintachai, Komwit Surachat, Ganyalak Chaimaha, Abdi Wira Septama, Duncan R. Smith

**Affiliations:** 1 School of Science, Walailak University, Thasala, Nakhon Si Thammarat 80161, Thailand; 2 Functional Materials and Nanotechnology Center of Excellence, Walailak University, Thasala, Nakhon Si Thammarat 80161, Thailand; 3 Department of Biomedical Sciences and Biomedical Engineering, Faculty of Medicine, Prince of Songkla University, Hat Yai, Songkhla 90110, Thailand; 4 Translational Medicine Research Center, Faculty of Medicine, Prince of Songkla University, Hat Yai, Songkhla 90110, Thailand; 5 Research Centre for Pharmaceutical Ingredients and Traditional Medicine, National Research and Innovation Agency (BRIN), South Tangerang 15314, Banten, Indonesia; 6 Institute of Molecular Biosciences, Mahidol University, Phutthamonthon, Nakhon Pathom 73170, Thailand

**Keywords:** multidrug-resistant *Acinetobacter baumannii*, phage, *Myoviridae*, *Phapecoctavirus*, biofilm, cell culture model

## Abstract

Multidrug-resistant *Acinetobacter baumannii* (MDR *A. baumannii*) is an emerging pathogen in the ESKAPE group. The global burden of antimicrobial resistance has led to renewed interest in alternative antimicrobial treatment strategies, including phage therapy. This study isolated and characterized a phage vB_AbaM_ ABPW7 (vABPW7) specific to MDR *A. baumannii*. Morphological analysis showed that phage vABPW7 belongs to the *Myoviridae* family. Genome analysis showed that the phage DNA genome consists of 148,647 bp and that the phage is a member of the *Phapecoctavirus* genus of the order *Caudovirales.* A short latent period and a large burst size indicated that phage vABPW7 was a lytic phage that could potentially be used in phage therapy. Phage vABPW7 is a high-stability phage that has high lytic activity. Phage vABPW7 could effectively reduce biofilm formation and remove preformed biofilm. The utility of phage vABPW7 was investigated in a human A549 alveolar epithelial cell culture model. Phage vABPW7 was not cytotoxic to A549 cells, and the phage could significantly reduce planktonic MDR *A. baumannii* and MDR *A. baumannii* adhesion on A549 cells without cytotoxicity. This study suggests that phage vABPW7 has the potential to be developed further as a new antimicrobial agent against MDR *A. baumannii*.

## 1. Introduction

Multidrug-resistant *Acinetobacter *baumannii** (MDR A. *baumannii*) is an important pathogen in the ESKAPE (*Enterococcus faecium*, *Staphylococcus aureus*, *Klebsiella pneumoniae* (*K. pneumoniae*), *A. baumannii*, *Pseudomonas aeruginosa* (*P. aeruginosa*), and *Enterobacter*) group, contributing to nosocomial and community-acquired infections. MDR *A. *baumannii** has become resistant to many antibiotics, including colistin, the last-resort antibiotic [1]. Infection of MDR A. *baumannii* in patients is associated with a variety of severe conditions such as bacteremia, pneumonia, ventilator-associated pneumonia, central nervous system infections, central line-associated bloodstream infections, catheter-associated urinary tract infections, wound infections, and surgical site infections, leading to increased morbidity and mortality, severe clinical outcomes, and longer lengths of stay at hospitals. In addition, one of the main concerns with MDR *A. *baumannii** is its ability to form biofilms, the community of multiple bacterial cells associated with either biotic or abiotic surfaces. During infection by MDR *A. *baumannii** in humans, MDR A. *baumannii* interacts and then adheres to epithelial cells. During the adherence, biofilm formation is induced, and bacterial adherence to the host cells is an essential step in MDR *A. *baumannii** colonization resulting in bacterial infection and subsequent pathogenesis [2,3,4]. Nowadays, *A. baumannii* has developed resistance to almost all of the available antibiotics [5]. Therefore, the development of alternative methods for MDR A. *baumannii* control is urgently required.

Bacteriophages or phages are bacterial viruses that enter and infect bacterial cells. The replication of the phage in host cells causes bacterial lysis and death, making them an alternative antibacterial treatment [6,7]. Moreover, phages might help to control the contaminations on the body surfaces of patients and healthcare workers, and environmental sites, according to the report on contaminations of the healthcare environment [8]. Phages have proven to be effective in the control of MDR bacteria [9]. Recently, a novel *Acinetobacter* phage AB02 was reported, and the phage showed therapeutic efficacy against MDR *A. *baumannii** [10]. Treatment with novel lytic phages (AB7-IBB1 and vABWU2101) reduced MDR *A. *baumannii** cells and biofilm formation [11,12]. Furthermore, a combination of phage and antibiotics has also been reported as efficacious in previous studies, including a combination of phage vB_AbaM-KARL-1 and antibiotics [13,14], a combination of phage vABWU2101 and tigecycline [12], and a combination of phage cocktail (containing Aba-1, Aba-2, Aba-3, Aba-4, and Aba-6) and several antibiotics [15]. Even though the effectiveness and safety of phages have been evaluated in various models such as artificial gut [16], *Galleria mellonella* [17,18], mouse [17], and clinical models [19], only a few studies have assessed the antibacterial activity of phages in cell culture models. *Clostridium* phage phiCDHS1 reduced the bacterial attachment and cell number in human colon tumorigenic HT-29 cells [20]. The intracellular *Escherichia coli* (*E*. *coli*) K1 in human epithelial cells was decreased by phage K1F [21]. The treatment of *Escherichia* phage P1 resulted in a significant reduction in *E. coli* in human colon HT29 cells [22]. However, to date, there have been no reports on the effectiveness of phage-specific MDR *A. *baumannii** in mammalian cells.

This study aimed to isolate a phage infecting MDR *A. *baumannii.** The biology of the phage was characterized, and the efficacy of the phage in vitro was determined, including antibiofilm activity. To the best of our knowledge, this is the first study on the antibacterial activity of the phage against MDR *A. *baumannii** in an A549 cell culture model.

## 2. Materials and Methods

### 2.1. Bacterial Strains and Growth Conditions

MDR A. baumannii (20 clinical isolates), E. coli (1 clinical isolate), K. pneumoniae (1 clinical isolate), methicillin-resistant Staphylococcus aureus (MRSA) (1 clinical isolate), and P. aeruginosa (1 clinical isolate) were kindly provided by the Natural Product Research Center of Excellence, Faculty of Science, Prince of Songkla University, Thailand. A. baumannii ATCC 17978 was included. After receiving the bacterial isolates, Gram-staining and biochemical tests (catalase and oxidase tests) were performed to confirm they were A. baumannii species. Routinely, bacterial isolates were cultured on tryptic soy agar plates (TSA; Becton, Dickinson and Company, Franklin Lakes, NJ, USA). After overnight incubation at 37 °C, bacterial colonies were transferred to 3 mL of sterile tryptic soy broth (TSB; Becton, Dickinson and Company, Franklin Lakes, NJ, USA). The bacterial suspension was incubated overnight at 37 °C with continuous shaking at 150 rpm. For the long-term storage of bacteria, bacterial isolates were mixed with sterile 40% (*v*/*v*) glycerol solution and were then stored at −80 °C.

### 2.2. Antibiotic Susceptibility of A. baumannii

The susceptibilities of *A*. *baumannii* strains to amikacin (30 µg), ceftazidime (30 µg), gentamicin (10 µg), imipenem (10 µg), colistin (10 µg), and tigecycline (15 µg) (Oxoid, Thermo Fisher Scientific, Waltham, MA, USA) were tested by the disc diffusion technique. 

### 2.3. Cell Culture

The human alveolar basal epithelial cell line A549 (ATCC Cat No. CCL-185) was provided by Professor Dr. Duncan Richard Smith, Institute of Molecular Biosciences, Mahidol University, Thailand. The cells were grown and maintained in Dulbecco’s modified Eagle’s medium (DMEM; Gibco, Invitrogen, Grand Island, NY, USA) supplemented with 10% heat-inactivated fetal bovine serum (Gibco, Invitrogen, Grand Island, NY, USA) at 37 °C with 5% CO_2_ in a humidified incubator.

### 2.4. Phage Enrichment and Isolation

The phage was enriched and isolated from wastewater samples collected from Thasala hospital, Nakhon Si Thammarat, Thailand. The MDR *A*. *baumannii* strain ABPW063 was employed as a host for phage isolation. The wastewater samples were centrifuged to remove the debris at 6000× *g*, 4 °C for 10 min. The supernatant was transferred to a new tube and then filtered. Ten milliliters of the filtrate were added to 10 mL of tryptic soy broth (TSB; Becton, Dickinson and Company, Franklin Lakes, NJ, USA), followed by the addition of MDR *A*. *baumannii.* The mixture was incubated at 37 °C for 24 h in a shaking incubator (150 rpm). The mixture was centrifuged to remove bacterial cells at 6000× *g*, 4 °C for 10 min. The supernatant was filtered through a syringe filter. A spot test was performed to detect phages. Briefly, MDR *A*. *baumannii* was mixed with top agar, and 10 µL of the filtrate was spotted onto the plate. The plates were incubated overnight at 37 °C and the lytic zone in the bacterial lawn, which indicates the presence of phages, was detected.

### 2.5. Phage Purification

A single plaque observed in the overlays was picked with a sterile pipette tip which was then soaked in an Eppendorf tube containing 500 μL SM buffer (0.1 M NaCl, 8 mM MgSO_4_. 7H_2_O, 50 mM Tris-HCl pH 7.5). After incubation at 4 °C overnight, the samples were centrifuged at 6000× *g* for 10 min at 4 °C and filtered through a sterile 0.22 μm filter. The filtrate was diluted with SM buffer in a series of ten-fold dilutions followed by the double-layer agar technique. The phage was purified five times to obtain purified phage.

### 2.6. Double-Layer Agar Technique

The supernatant was diluted with SM buffer by a series of ten-fold dilutions. Two hundred microliters of the phage-diluted solution was mixed with 200 µL of log-phase MDR *A. baumannii* culture. The mixture was mixed with top agar (0.75% agar in TSB) and then quickly poured onto a TSA plate. Plaques were counted after incubation overnight at 37 °C.

### 2.7. Phage Stock Preparation and Phage Enumeration

To amplify a phage stock, the phage supernatant was diluted with SM buffer by a series of ten-fold dilutions. The dilution (200 μL) was mixed with exponential-phase bacterial culture (200 μL) in top agar. The mixture was poured on a TSA plate and incubated at 37 °C overnight. The phage was eluted by adding 5 mL SM buffer onto semi-confluent plates. The supernatant was pooled and centrifuged to remove the bacterial cells at 6000× *g* for 20 min at 4 °C. The supernatant was filtered through a sterile 0.22 μm filter and then concentrated using an Amicon^®^ Ultra-15 centrifugal filter units, Ultracel 100 kDa membrane (Merck Millipore, Burlington, MA, USA) [23]. The supernatant was collected as a phage stock and kept at 4 °C until used. The phage titer was determined using the double-layer agar technique as calculated as a plaque-forming unit per mL (PFU/mL).

### 2.8. Phage Morphological Characterization

A morphological observation of the phage was performed by transmission electron microscopy (TEM) as described previously [18]. The phage stock was dropped onto a carbon-coated copper grid and negatively stained with 2% (*v*/*v*) uranyl acetate (pH 6.7). The phage was visualized using a JEM 2010, JEOL electron microscope (JEOL USA, Inc., Peabody, MA, USA) at an operating voltage of 160 kV. The phage classification based on the morphological characteristics was determined according to the standard phage classification method [24].

### 2.9. Phage Host Range Determination

The standard spot test was performed to evaluate the lytic activity of the phage as previously described [18]. The host range of the phage was determined against 20 clinically isolated MDR *A. baumannii*, *A. baumannii* ATCC 17978, *E. coli*, *K. pneumoniae*, MRSA, and *P. aeruginosa* strains. Two hundred microliters of exponential-phase clinical bacterial isolates was mixed with top agar and poured onto a TSA plate. Ten microliters of the diluted phage stock containing 10^4^ PFU/mL was dropped on the surface of the top agar inoculated with 200 μL of each bacterial culture. The plates were left to dry and then incubated overnight at 37 °C. The presence of a clear zone in the bacterial lawn was regarded as the lytic activity of the phage, while the absence of the clear zone indicated no lytic activity. Experiments were undertaken independently in duplicate.

### 2.10. The Efficiency of Plating of Phage (EOP)

The phage lytic activity was confirmed by calculating the EOP as previously described [25]. The spot-test-positive strains were analyzed by the EOP assay. The phage was diluted with SM buffer in a series of ten-fold dilutions, and 100 µL of the diluted phage was mixed with 200 µL of each log-phase MDR *A. baumannii* isolate. The mixtures were incubated at room temperature for 20 min, followed by the double-layer agar technique. After incubation overnight at 37 °C, the plaques were counted. The EOP value was calculated as the ratio of the titer of the phage on a test isolate to the titer of the phage on the host bacterium. Experiments were undertaken independently in duplicate.

### 2.11. Phage Adsorption Rate Assay

The attachment rate of the phage to MDR *A. baumannii* was measured following a previously reported methodology [25]. The phage was mixed with MDR *A. baumannii* at an MOI of 0.1, followed by incubation at 37 °C without shaking. Samples were taken every minute for the first 10 min, after which samples were taken every 5 min from 10 to 30 min post-infection. The samples were immediately added to pre-chilled TSB supplemented with chloroform. The phage titer was then determined by double-layer agar assay. Experiments were undertaken independently in duplicate with the duplicate plaque assay. The adsorption rate constant, *k*, in mL/min, was calculated by following the equation as previously described [26].

*k* = 2.3 log Po   B*t*      P

where *k* is the adsorption rate constant (mL/min); B is the bacterial cell concentration; *t* is the time; Po is the stating titer; P is the final titer.

### 2.12. One-Step Growth Curve

The latent phage period and burst size were inferred from a one-step growth curve as previously described [18,27]. Briefly, exponential-phase MDR *A. baumannii* was centrifuged at 6000× *g* for 20 min at 4 °C. The supernatant was removed, and the bacterial pellet was re-suspended in TSB. The bacteria were infected with the phage at an MOI of 0.1. To allow for the absorption of the phage into bacterial cells, the mixture of bacteria and phage was incubated for 20 min at 37 °C. After centrifugation at 6000× *g* for 20 min at 4 °C to remove any unabsorbed phage, the pellet was re-suspended in TSB and then incubated at 37 °C with shaking at 150 rpm. Samples were taken every 10 min for 120 min, and the phage was titered by the soft-agar overlay method. The time between phage adsorption and cell lysis was the latent period. The burst size and the average number of phages released per bacterium was calculated from the ratio of the total number of the released phage progeny at the end of one growth cycle to the initial count of infected bacterial cells. Experiments were undertaken independently in duplicate with the duplicate assay [28].

### 2.13. Killing Activity of the Phage

The effect of the phage against MDR *A. baumannii* growth was assessed following the previously established methodology [29]. MDR *A. baumannii* was cultured at 37 °C overnight, and the concentration of bacterial cells was adjusted to 1 × 10^8^ colony-forming units per mL (CFU/mL). MDR *A. baumannii* was infected with phage at MOIs of 0.1, 1, and 10. The samples were incubated at 37 °C with constant shaking. The supernatant was taken every hour for 8 h to measure the bacterial growth at 600 nm. Experiments were undertaken independently in duplicate with the duplicate assay [30].

### 2.14. Scanning Electron Microscopy (SEM)

The effects of the phage on MDR *A. baumannii* cells were visualized under SEM. MDR *A. baumannii* cells were infected with the phage at an MOI of 1 and then incubated at 37 °C for 4 h. The samples were centrifuged at 6000× *g* for 10 min. The supernatant was removed, and the bacterial pellets were washed twice with 1 mL of PBS. The bacterial cells were re-suspended in 200 µL of PBS, and the bacterial suspension was dropped onto glass slides. After cell fixation with 2.5% glutaraldehyde in 0.1 M PBS, the cells were washed twice with PBS. The cells were dehydrated in a series of ethanol solutions (20%, 40%, 60%, 80%, and 100%) after which the bacterial cells were dried in a critical point dyer and then coated with gold. The ultrastructure of bacterial cells was observed under a field emission SEM (FE-SEM) (Merlin VP Compact, Carl Zeiss AG, Oberkochen, Germany).

### 2.15. Effect of Temperatures, pH Values, Glycerol, and UV Radiation on the Phage Stability

The effects of different temperatures, pH values, and UV radiation on the phage stability were measured according to the previously described methods [18]. The phage stock was prepared and diluted in the SM buffer to 10^8^ PFU/mL. The bacteriophage in the SM buffer was incubated at different temperatures (4 °C, 25 °C, 37 °C, 50 °C, 60 °C, and 70 °C) for 2 h. After incubation at specific temperatures, the samples were cooled in an ice-water bath, and the phage viability was determined by the double-layer agar method. The phage incubated at 4 °C was used as a control. For the ability of the phage to tolerate varying pH values, the phage was incubated in SM buffers ranging from pH 1 to pH 14 for 2 h, after which the samples at varying pH levels were neutralized to pH 7, and the phage viability was measured by the double-layer agar method. The phage incubated in the SM buffer at pH 7 was used as a control. The phage stability in glycerol stock was evaluated. The phage was mixed with glycerol in the SM buffer. The final concentrations were 25% (*v*/*v*) and 50% (*v*/*v*) glycerol [23]. After incubation at −20 °C and −80 °C for 30 days, the viability of the phage was determined by the double-layer agar method. The stability of the phage was also examined under UV radiation. The phage was added into open Petri dishes and incubated on ice. The plates were positioned approximately 30 cm away from a UV light source. The samples were collected at 10 min intervals for 1 h, and the phage titer was determined by the double-layer agar. Experiments were undertaken independently in duplicate with the duplicate plaque assay.

### 2.16. Phage Whole-Genome Sequencing

The phage whole-genome de novo sequencing was undertaken commercially (Macrogen Inc., Seoul, South Korea) on the Illumina sequencing platform. The phage DNA was extracted, and the quality was verified by the Picogreen method using Victor 3 fluorometry (Invitrogen, Grand Island, NY, USA). The library was prepared using the TruSeq Nano DNA library preparation kit (Illumina Inc., San Diego, CA, USA). After checking the library quality, the library was sequenced, and the quality of raw sequence data from high throughput sequencing pipelines was evaluated by FastQC (version 0.11.5), a quality control tool. The results from FastQC were assembled de novo into a contig by SPAdes de novo (version 3.13.0) with the k-mer parameter. Prokka (version 1.12) was used to predict the locations of protein-coding sequences, tRNA genes, tmRNA genes, and rRNA genes. BLAST and EggNOG were used to annotate the functions of genes. Blastx was performed against standard databases with an E-value cutoff of 0, a query coverage cutoff of 80%, and an identity cutoff of 80%. The genome map was constructed on the CGview server.

The relationship of the phage to other phages in the NCBI database was determined by a Blastn search with an E-value cutoff of 0, and an identity cutoff of 70%. The outgroup was *Staphylococcus* phage JD419 (QOI66744). All pairwise comparisons of the nucleotide sequences were analyzed by the Genome-BLAST Distance Phylogeny approach under settings recommended for prokaryotic viruses [31,32,33]. The intergenomic distances were used to infer a balanced minimum evolution via FASTME and SPR post-processing [34]. Branch support was identified from 100 pseudo-bootstrap replicates each. The tree was constructed by FigTree [35], and the taxon level was analyzed by the OPTSIL program [36], using the recommended clustering thresholds [33], with an F value of 0.5 [37]. The genomic synteny was evaluated by ViPtree [38]. EMBOSS Stretcher was used to evaluate the sequence identity.

### 2.17. Phylogenetic Analysis of the Tail Fiber Gene

The tail fiber gene was selected to investigate the genetic relationships using the maximum-likelihood phylogenetic tree and the Jones–Taylor–Thornton matrix-based model based on the amino acid sequence alignment. The gene was compared with other gene sequences in the NCBI database by Blastx [39]. The multiple sequence alignment was performed with MUSCLE, and the maximum-likelihood phylogenetic tree was constructed in MEGA-X using 1000 bootstrap replicates. The tail fiber of *Staphylococcus* phage JD419 (QOI66744) was used as an outgroup.

### 2.18. Biofilm Assay

The ability of the phage to prevent biofilm formation and to remove biofilm was assessed according to the methods described previously [18]. The biofilm biomass and the viable cell numbers were determined by crystal violet staining and colony counting, respectively. The biofilm biomass and cell viability were evaluated in parallel. To assess the efficacy of phage on biofilm formation, MDR *A. baumannii* was cultured and diluted with TSB. One hundred microliters of MDR *A. baumannii* was added into a flat-bottomed 96-well microtiter plate (10^7^ CFU/well). Subsequently, 100 μL of diluted phage (10^1^–10^8^ PFU/well) was added to the wells, and the plates were incubated without agitation at 37 °C for 24 h. For the disruption of preformed biofilm, bacteria were added into a flat-bottomed 96-well microtiter plate and incubated at 37 °C for 24 h without shaking to allow for biofilm formation. At the indicated time point, the supernatant was removed. The biofilm was washed twice with PBS, and 100 µL of the phage (10^1^–10^8^ PFU/well) was added to the wells. The plates were incubated at 37 °C for 24 h. At the indicated time points, the plates were washed twice with PBS and then air-dried. Two hundred microliters of 0.1% crystal violet was added to stain the biofilm biomass. After incubation at room temperature for 30 min, the excess stain was washed away with PBS, and 200 μL of absolute ethanol was added to solubilize the biofilm. The biofilm biomass was measured at 595 nm using a standard microplate absorbance reader. To determine biofilm viable cell numbers, the supernatant was removed, and the biofilm was washed twice to remove planktonic bacteria with PBS. The biofilm was re-suspended in 100 µL of PBS by vigorous pipetting. The supernatant was serially diluted in PBS. Ten microliters of the supernatant were dropped onto TSA plates and air-dried. The colonies were counted after incubation at 37 °C overnight. Experiments were undertaken independently in triplicate with the duplicate assay.

### 2.19. Cytotoxicity of the Phage and MDR A. baumannii

The cytotoxicity of the phage and MDR *A. baumannii* towards A549 lung epithelial cells was examined using the MTT (3-(4,5-dimethylthiazol-2-yl)-2,5-diphenyltetrazolium bromide) cell proliferation assay. The cells were grown in 96-well tissue culture plates at 37 °C with 5% CO_2_ until the cells reached approximately 90% confluence. The medium was discarded, and cells were then washed with PBS. The cells were incubated with 200 µL of the phage (MOIs of 0.01 to 100) or MDR *A. baumannii* (MOIs of 0.01 to 100) diluted in cell culture medium. After incubation under standard conditions for 24 h, the medium was removed, and the cells were washed twice with PBS. One hundred microliters of complete fresh medium were added to the wells followed by the MTT solution. After incubation under standard conditions for 4 h, DMSO was added into the well to solubilize the water-insoluble formazan. Absorbance was measured at a wavelength of 570 nm using a standard microplate absorbance reader. Cells incubated with only a combination of cell culture media and SM buffer (or only cell culture media) and cells incubated with 5% DMSO were included as negative and positive controls, respectively. The experiments were undertaken independently in triplicate with the duplicate assay.

### 2.20. Phage Adsorption to A549 Cells

The adsorption of phage to A549 cells was undertaken as described by others [20]. Briefly, A549 cells were seeded in 6-well tissue culture plates, and the cells were grown under standard conditions until the cells reached approximately 90% confluence. The cell culture medium was removed, and the cells were washed twice with PBS. The cells were incubated with the phage at an MOI of 1, followed by incubation under standard conditions. The supernatant was collected every 30 min for 2 h. The phage titer was measured by counting the plaques using a double-layer plaque assay. The experiments were undertaken independently in duplicate with the duplicate plaque assay.

### 2.21. Screening of Antibacterial Activity of the Phage under a Cell Culture Model

The phage efficacy in reducing an MDR *A. baumannii* infection under the cell culture model was determined following the methodology in a previous report [22]. The cell viability under each condition was assessed in parallel with an MTT cell viability assay. Briefly, A549 cells were seeded in 96-well tissue culture plates under standard conditions and then cultured until the cells reached approximately 90% confluence. The cell culture medium was discarded, and the cells were then washed with PBS. The cells were infected with MDR *A. baumannii* at an MOI of 1. After incubation for 2 h, the cells were washed twice with PBS and then incubated with the phage at MOIs of 0.01 to 100 under standard conditions for 2 h. The medium was removed, the cells were washed twice with PBS, and complete medium was added; the cells were then cultured under standard conditions. At 24 h post-incubation, the supernatant was removed and replaced with 100 μL serum-free media containing 0.2% resazurin. The plates were incubated under standard conditions for 3 h and the fluorescence was taken at 530/590 nm to measure the reduction in bacteria. On the parallel plates, the MTT assay was undertaken as previously described. The experiments were undertaken independently in duplicate.

### 2.22. Kinetics of the Antibacterial Activity of the Phage against Planktonic Bacteria under a Cell Culture Model

The anti-planktonic property of phage vABWU7 under the cell culture model was assessed, and the number of planktonic bacteria and free phage production were evaluated at time intervals for 6 h. A549 cells were cultured in 6 well tissue culture plates under standard conditions and then grown until the cells reached approximately 90% confluence. Overnight cultures of MDR *A. baumannii* were centrifuged at 6000× *g* for 10 min. The supernatant was removed, and the bacterial pellet was washed three times with PBS. The bacterial cells were resuspended in DMEM and then adjusted to a final OD600 of 1.0 before proceeding with the bacterial adhesion assays. After removing the cell culture medium, the A549 cells were washed with PBS and incubated with MDR *A. baumannii* at an MOI of 1 and phage at MOIs of 5 and 10. The cells were incubated under standard condition, and the supernatant was collected at 0, 1, 2, 4, and 6 h post-incubation. The production of planktonic bacterial cells was measured by counting the colony-forming units after plating cultures. The supernatants were serially diluted ten-fold and then spread onto TSA plates. The production of free phage was determined by counting plaques using a double-layer agar method. At 6 h post-incubation, the viability of A549 cells was assessed by a trypan blue exclusion test. The kinetics of the antibacterial activity of the phage against planktonic bacteria under the model without cells were evaluated in parallel. The experiments were undertaken independently in duplicate with duplicate plaque assay.

### 2.23. Effect of the Phage on the Bacterial Adhesion to Mammalian Cells

The effect of the phage on the adhesion of MDR *A. baumannii* to A549 cells was evaluated as previously described [40,41,42,43]. The experiments were undertaken as described above in the antibacterial activity of the phage against planktonic bacteria assays. The experiments were divided into 2 conditions: prophylactic and therapeutic treatments. For the prophylactic treatment, the cells were incubated with the phage for 2 h, followed by MDR *A. baumannii* infection for 2 h. For the therapeutic treatment, the cells were infected with MDR *A. baumannii* for 2 h and then treated with the phage for 2 h. The cells were incubated under standard conditions, and the bacterial number was assessed at 0, 1, 2, 4, and 6 h post-incubation. The supernatant was discarded at the indicated time points to remove non-adherent bacteria. The cells were washed three times with PBS to remove non-adherent extracellular bacteria and phage. Subsequently, the cells were treated with 1 mL of PBS containing 0.1 mM EDTA and incubated at 37 °C for 10 min, followed by vigorous pipetting. The supernatant was serially diluted ten-fold and then spread onto TSA plates. The viable bacterial cells were determined by counting colonies formed after overnight incubation at 37 °C. In addition, the cell viability was also determined in parallel by the trypan blue exclusion test. The experiments were undertaken independently in duplicate with the duplicate assay.

### 2.24. Ultrastructure of the Adhesion of Bacteria to the Cells

The effects of the phage on MDR *A. baumannii* that attached to A549 cells were determined following the protocol as described above, and the ultrastructure of the bacteria on the cell surface was visualized. Briefly, the phage at an MOI of 5 were added to the cells 2 h before MDR *A. baumannii* infection (at an MOI of 1) in prophylactic phage administration, while the cells were infected with MDR *A. baumannii* cells (an MOI of 1) before the phage treatment (at an MOI of 5) in the therapeutic treatment. The cells were incubated under standard condition for 4 h and then washed twice with PBS. The cells were fixed for SEM analysis following the protocol as described above. The ultrastructure of bacterial cells attached to A549 cells was observed under an FE-SEM (Merlin VP Compact, Carl Zeiss AG, Oberkochen, Germany).

### 2.25. Statistical Analyses

The GraphPad Prism program, version 5 (GrapPad Software, Available online: https://www.graphpad.com/scientific-software/prism/ (accessed on 1 September 2022)) was used to analyze all data. Statistical significance analysis was undertaken by unpaired T-test using the GraphPad Prism, *p* < 0.05 for significance.

### 2.26. Nucleotide Sequence Accession Number

The phage genome was deposited in the GenBank database with the accession number OP562383.

## 3. Results

### 3.1. Antibiotic Susceptibility of A. baumannii Isolates

All *A. baumannii* strains were resistant to amikacin, ceftazidime, gentamicin, and imipenem (Supplementary Table S1). Four (4/20 strains, 20%) and three (3/20 strains, 15%) *A. baumannii* strains were sensitive and intermediate to tigecycline, respectively. All strains were sensitive to colistin. *A. baumannii* strains were resistant to at least one agent in three or more chemical classes of antibiotic [44], indicating that all *A. baumannii* strains were MDR *A. baumannii.*

### 3.2. Phage Isolation

**Figure 1:**
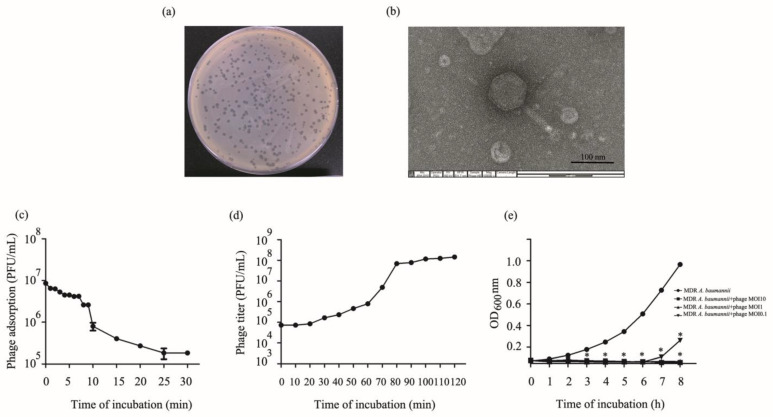
Isolation and biological characterization of phage vABPW7. (**a**) Plaques of phage vABPW7 in an MDR *A*. *baumannii* lawn. (**b**) Transmission electron micrograph of phage vABPW7. (**c**) Adsorption rate of phage vABPW7 to the host bacterial strain. (**d**) One-step growth curve showing the latent period and burst size of phage vABPW7. (**e**) Reduction in bacterial growth by phage vABPW7 at different MOIs. Experiments were undertaken independently in duplicate with duplicate assay. The data show the mean ± SEM (* *p* value < 0.05).

A phage was isolated from hospital wastewater samples, and MDR *A. baumannii* ABPW063 was used as the host strain. The phage formed clear plaques of 1–2 mm diameter with a halo on an MDR *A. baumannii* lawn (Figure 1a). The phage morphology was investigated by electron microscopy. The phage has an icosahedral head with a diameter of 94.67 ± 8.17 nm from vertex to vertex and a long contractile tail of length 103.6 ± 7.8 nm (n = 3) (Figure 1b). Based on morphological similarities, the isolated phage likely belongs to the *Myoviridae* family. The phage was named phage vB_AbaM_ABPW7 or phage vABPW7 according to the binomial nomenclature of bacteria viruses [45].

### 3.3. Phage Host Range and EOP

The host range of *Acinetobacter* phage vABPW7 was determined by spot tests against 20 clinically isolated MDR *A. baumannii*, *A. baumannii* ATCC 17978, *E. coli*, *K. pneumoniae*, MRSA, and *P. aeruginosa* strains. The results showed that phage vABPW7 lysed 45% of MDR *A. baumannii*, indicating that phage vABPW7 might be suitable for controlling an MDR *A. baumannii* infection. *A. baumannii* ATCC 17978, *E. coli*, *K. pneumoniae*, MRSA, and *P. aeruginosa* were found to be insensitive to the phage. The ability of phage vABPW7 to lyse MDR *A. baumannii* was also assessed by an EOP assay. The EOP value is the ratio of lysis plaques produced in each susceptible strain divided by the number of plaques produced in a host cell. The EOP was performed on 9 MDR *A. baumannii* isolates that were the spot-test-positive strains. EOP is classified as high (EOP ≥ 0.5), moderate (0.5 > EOP ≥ 0.1), low (0.1 > EOP > 0.001), and no activity (EOP ≤ 0.001). Three, four, and two isolates were found to exhibit a high EOP value (EOP = 0.61–1), moderate EOP value (EOP = 0.1–0.4), and low EOP value (EOP = 0.01–0.04), respectively (Table 1).

### 3.4. Phage Adsorption

The kinetic of phage adsorption to MDR *A. baumannii* cells was determined. The phage particles were adsorbed into the host MDR *A. baumannii* ABPW063 isolate by approximately 90% within 10 min. At 15 min post-incubation, the adsorption reached more than 95% (Figure 1c). The adsorption efficiency of phage vABPW7 to the host strain was derived as an adsorption rate constant *k* value of 1.19 × 10^−8^ mL/min.

### 3.5. One-Step Growth Curve

The infection dynamics of phage vABPW7 was determined by a one-step growth curve assay. The latent period was about 20 min (Figure 1d). The calculated burst size of the phage, the ratio of the free phage to the number of bacteria initially infected, was approximately 145.25 ± 5.3 PFU/cell.

### 3.6. Lytic Activity of Phage vABPW7 against MDR A. baumannii

To evaluate the bacteriolytic effect of phage vABPW7 against MDR *A. baumannii*, a killing curve was determined with different MOIs of the phage. MDR *A. baumannii* was infected with the phage at MOIs of 0.1, 1, and 10, and the supernatant was collected every hour for 8 h. The bacterial growth was determined by OD600 measurement. The results showed that the turbidity of uninfected MDR *A. baumannii* increased continuously (Figure 1e). For the phage treatments, the turbidity of phage-infected MDR *A. baumannii* at MOIs of 0.1, 1, and 10 decreased gradually at 1 h post-infection. At 2 h.p.i., the turbidity of phage-infected MDR *A. baumannii* was significantly less than that in the non-phage-infected control. The turbidity of MDR *A. baumannii* infected with phage vABPW7 at MOIs of 1 and 10 was significantly reduced at 8 h post-incubation. However, the turbidity of MDR *A. baumannii* infected with phage vABPW7 at an MOI of 0.1 started to increase from 7 h post-incubation.

### 3.7. Structural Details of Bacterial Cells after Phage vABPW7 Infection

**Figure 2:**
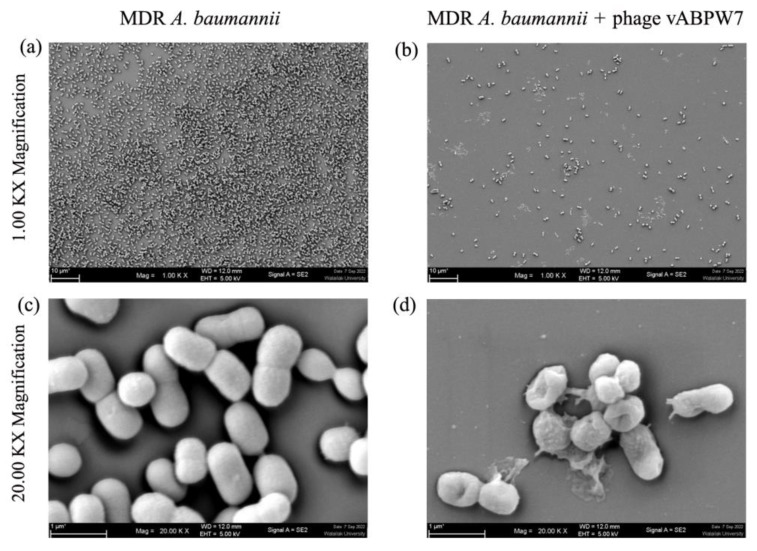
Morphology of MDR *A. baumannii* cells under FE-SEM. (**a**,**c**) Untreated MDR *A. baumannii* cells. (**b**,**d**) MDR *A. baumannii* infected with phage vABWU7. The cells were observed at magnifications of ×1000 and ×20,000.

The morphology of MDR *A. baumannii* cells after phage vABPW7 treatment was observed under SEM. The number of bacterial cells in the control bacteria without phage treatment was higher than in the phage treatment group (Figure 2a,b). The morphology of MDR *A. baumannii* cells was a coccobacillus. Uninfected MDR *A. baumannii* cells displayed a smooth and intact surface (diameter 0.64 ± 0.04 μm and length 1.03 ± 0.12 μm (n = 3)) (Figure 2c). MDR *A. baumannii* infected with phage vABPW7 showed a morphological change (diameter 0.56 ± 0.04 μm and length 0.85 ± 0.04 μm (n = 3)). The phage induced blisters and pores on the bacterial cell membrane. MDR *A. baumannii* cells had burst, with cell lysis, leading to cell death (Figure 2d).

### 3.8. Stability of Phage vABPW7 under Various Temperatures, pH Values, Glycerol Storage, and UV Radiation

**Figure 3:**
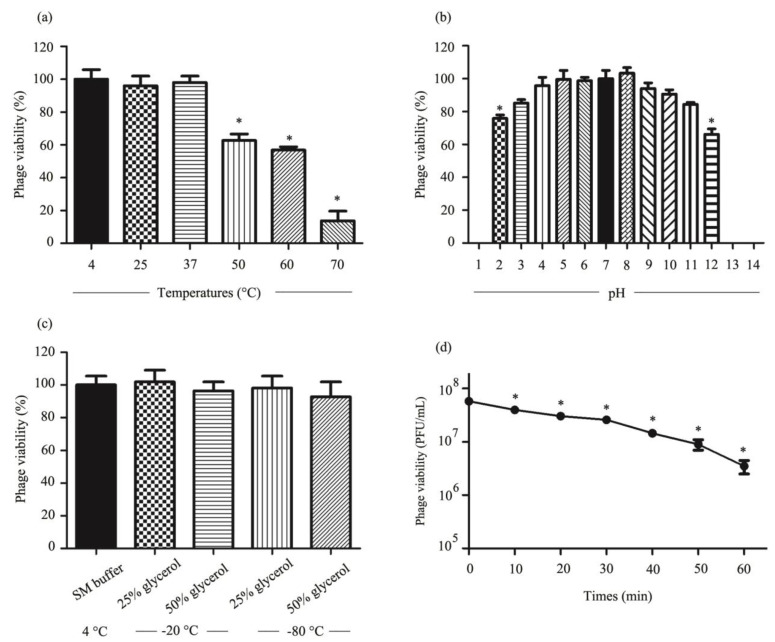
Stability of phage vABPW7 under different conditions. (**a**) Thermal stability of phage vABPW7 incubated at different temperatures for 2 h. (**b**) pH stability of phage vABPW7 incubated at different pHs for 2 h. (**c**) Stability of phage vABPW7 under varied concentrations of glycerol at −20 °C and −80 °C for 30 days (**d**) UV stability of phage vABPW7. Experiments were undertaken independently in duplicate with duplicate assay. The data show the mean ± SEM (* *p* value < 0.05).

To assess the stability of phage vABPW7, thermal stability tests were performed at different temperatures from 4 to 70 °C. There was no significant change in phage stability after incubation between 4 to 37 °C (Figure 3a). The phage titer was significantly decreased after incubation at 50, 60, and 70 °C. The phage stability under different pH (pH 1 to 14) was assessed. The phage had a stable titer between pH 3 and pH 11 (Figure 3b). The lytic activity of phage vABPW7 significantly decreased at pH 2 and 12. There was no lytic activity at pH 1, 13, and 14. The effects of different concentrations of glycerol on the stability of phage vABPW7 were also evaluated after incubation at −20 and −80 °C. There were no significant changes in phage viability with 25% (*v*/*v*) and 50% (*v*/*v*) glycerol after storage at −20 and −80 °C for 30 days when compared with the stability of the phage in SM buffer at 4 °C, as a control (Figure 3c). The infective ability of phage vABPW7 under UV radiation was evaluated. A significant reduction in phage titer was observed at 10 min post-exposure. After phage exposure to UV for 60 min, the phage titer was significantly decreased to about 6.16% (Figure 3d).

### 3.9. Whole-Genome Sequencing and Analysis

**Figure 4:**
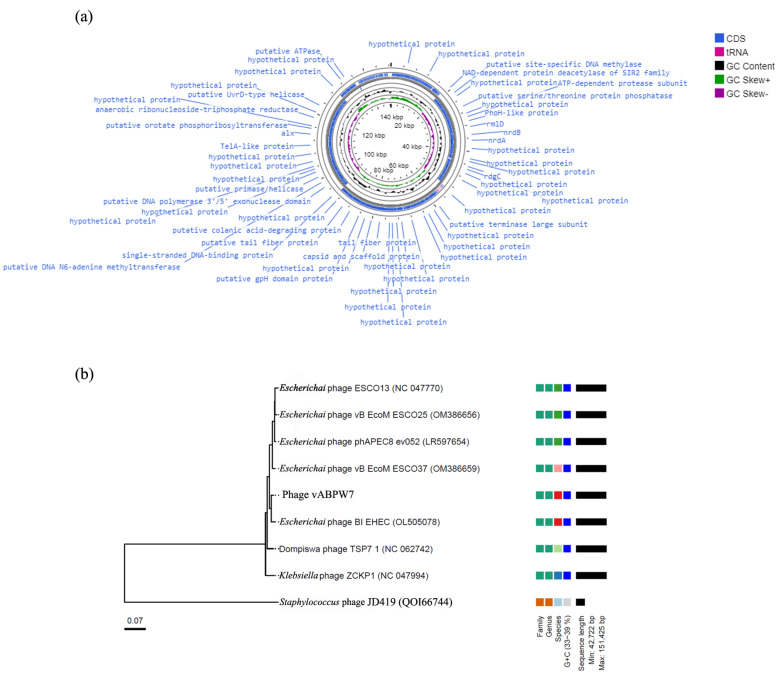
Phage genome characterization. (**a**) Circular genome map of phage vABPW7. The graphical map of the genome was generated using GC viewer server^Beta^. (**b**) Phylogenetic tree analysis of the whole-genome sequence of phage vABPW7 and related phages. The phylogeny was assessed by the VICTOR program. *Staphylococcus* phage JD419 (QOI66744) was used as an outgroup.

The whole genome of phage vABPW7 was sequenced using the Illumina sequencing system, and the phage sequences were de novo assembled and annotated. The genome of the phage was 148,647 bp in size, and the G+C content was 39.04% (Figure 4a). Genomic analysis predicted 274 genes and Blastx was used to determine the likely functions of predicted genes. The phage genome includes 71 proteins of a known putative function and 203 hypothetical proteins (Supplementary Table S2). Nine coding sequences encoded 9 tRNAs. There were no rRNA, tmRNA, virulence genes, or lysogeny-related genes in the genome of phage vABPW7. The coding sequences with known putative function were grouped into four main clusters: 38 coding sequences with DNA replication/modification functions, 20 coding sequences with structural protein functions, 4 coding sequences associated with host infection, and 9 coding sequences encoding tRNAs. The genome was also annotated using EggNOG. The results indicated 287 predicted genes, and the functions of the predicted genes corresponded with the Blastx analysis (Supplementary Table S3).

To evaluate the relationship of phage vABPW7 and other phages on a nucleotide level, the whole-genome sequence of phage vABPW7 was searched against the Blastn database and the data were further analyzed by VICTOR, a genome-based phylogeny and classification of prokaryotic viruses program. Phage vABPW7 was the most closely related to *Escherichia* phage BI-EHEC (OL505078.1) (Figure 4B). Other related phages to phage vABPW7 were *Escherichia* phage vB_EcoM_ESCO37 (OM386659.1), *Escherichia* phage phAPEC8_ev052 (LR597654.1), *Escherichia* phage vB_EcoM_ESCO25 (OM386656.1), *Escherichia* phage ESCO13 (NC_047770.1), Dompiswa phage TSP7_1 (NC_062742.1) and *Klebsiella* phage ZCKP1 (NC_047994.1), respectively. *Staphylococcus* phage JD419 (QOI66744) was used as the outgroup. The sequencing results showed that phage vABPW7 was a member of the genus *Phapecoctavirus*, subfamily *Stephanstirmvirinae*, class *Caudoviricetes,* and order *Caudovirales.*

**Figure 5:**
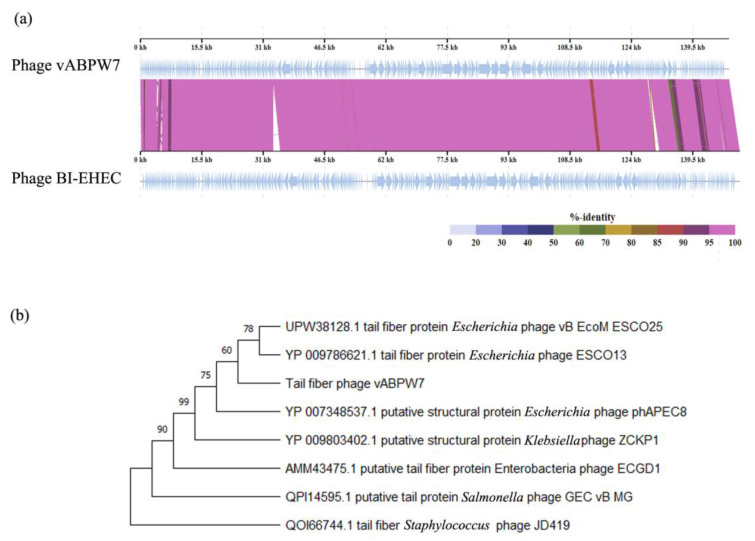
Genomic analysis of phage vABPW7. (**a**) A synteny plot displaying the genome structure of phage vABPW7 compared to *Escherichia* phage BI-EHEC, which is the most closely related phage to phage vABPW7. (**b**) A phylogenetic tree of the tail fiber protein of phage vABWU7. The multiple amino acid sequences were aligned by MUSCLE and the maximum-likelihood phylogenetic tree (JTT matrix-based model) was constructed in MEGA-X using 1000 bootstrap replicates. The tail fiber of *Staphylococcus* phage JD419 (QOI66744) was used as an outgroup.

The genomic synteny of phage vABPW7 and the closest phage, *Escherichia* phage BI-EHEC, was analyzed by ViPtree. A high identity of both genomes was observed across the majority of the genomes, although regions of lower identity were seen (Figure 5a). EMBOSS Stretcher was used for the quantitation of genomic identity based on the previous report [46]. The results showed that both phages shared 95.1% genome identity, suggesting that phage vABPW7 was the same species as *Escherichia* phage BI-EHEC in the genus *Phapecoctavirus*.

### 3.10. Phylogenetic Tree Analysis of the Tail Fiber Protein

To analyze the relationship of phage vABPW7, a phylogenetic tree analysis was carried out with the tail fiber protein encoded by ORF159. The nucleotide sequence of the gene was searched with Blastx against the NCBI database with an E-value cutoff of 0, a query coverage cutoff of 80%, and an identity cutoff of 70%. The phylogenetic tree results showed that phage vABPW7 shared a common clade ancestor with the tail fiber protein of *Escherichia* phage ESCO13 (YP_009786621.1, 99.37% identity) and *Escherichia* phage vB B_EcoM_ESCO25 (UPW38128.1, 99.37% identity) (Figure 5b). The tail fiber protein of phage vABPW7 also showed high similarity with a putative structural protein of *Escherichia* phage phAPEC8 (YP_007348537.1, 99.27% identity), a putative structural protein of *Klebsiella* phage ZCKP1 (YP_009803402.1, 98.75% identity), a putative tail fiber protein of *Enterobacteria* phage ECGD1 (AMM43475.1, 83.19% identity), and a putative tail protein of *Salmonella* phage GEC vB MG (YP_009803402.1, 73.89% identity), respectively. The tail fiber of *Staphylococcus* phage JD419 (QOI66744) was used as an outgroup.

### 3.11. Efficacy of Phage vABPW7 against MDR A. baumannii Biofilm

**Figure 6:**
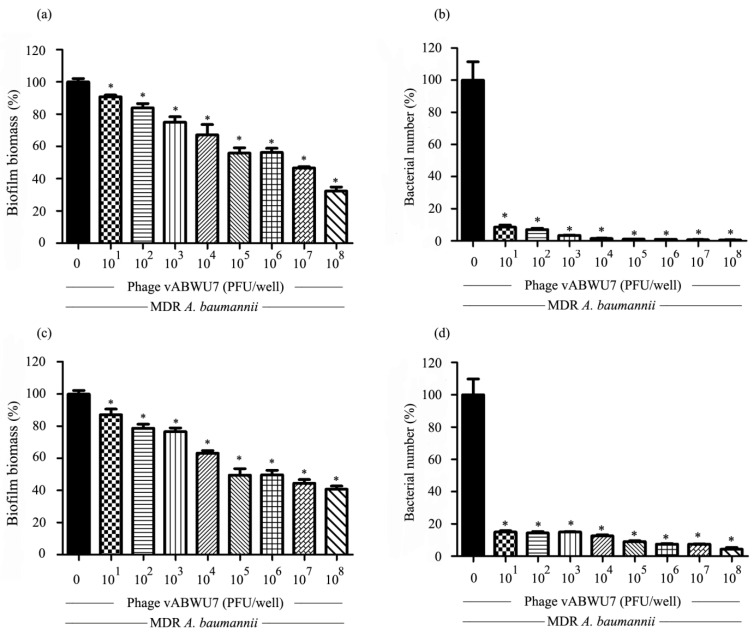
Efficacy of phage vABWU7 to prevent biofilm formation and remove preformed biofilm. The efficacy of phage vABWU7 to prevent biofilm formation was evaluated. The biofilm biomass (**a**) and bacterial cell viability (**b**) were determined by the crystal violet assay and colony counting method, respectively. The ability of phage vABWU7 to remove preformed biofilm was investigated by quantification of biofilm biomass (**c**) and cell viability in biofilm formation (**d**). Experiments were undertaken independently in triplicate with duplicate assay. The data show the mean ± SEM (* *p* value < 0.05).

The ability of phage vABPW7 to prevent biofilm development or disrupt matured biofilm was investigated by crystal violet staining-based biofilm biomass quantification and bacterial viability enumeration. To prevent biofilm formation, MDR *A. baumannii* was incubated with phage vABPW7 at various concentrations. Phage vABPW7 at 10^1^ to 10^8^ PFU/wells were significantly effective in preventing biofilm formation by reducing approximately 9.25 to 67.27% biofilm biomass, and 91.5 to 99.4% (1.2 to 2.05 log) of viable cells compared to a control, not treated with phage (Figure 6a,b). The efficacy of phage vABPW7 to remove preformed biofilm was investigated. The biofilm was formed followed by the treatment of phage vABPW7 at various concentrations. The preformed biofilm significantly removed both biofilm biomass and biofilm cell viability by approximately 12.79 to 59.27%, and 85.1 to 95.6% (0.8 to 1.2 log) after the treatment of phage vABPW7 at 10^1^ to 10^8^ PFU/well, respectively (Figure 6c,d). The significant difference of each phage concentration was analyzed by comparing the phage-treated group with a control that was not treated with phage.

### 3.12. Cytotoxicity of Phage vABPW7 and MDR A. baumannii

**Figure 7:**
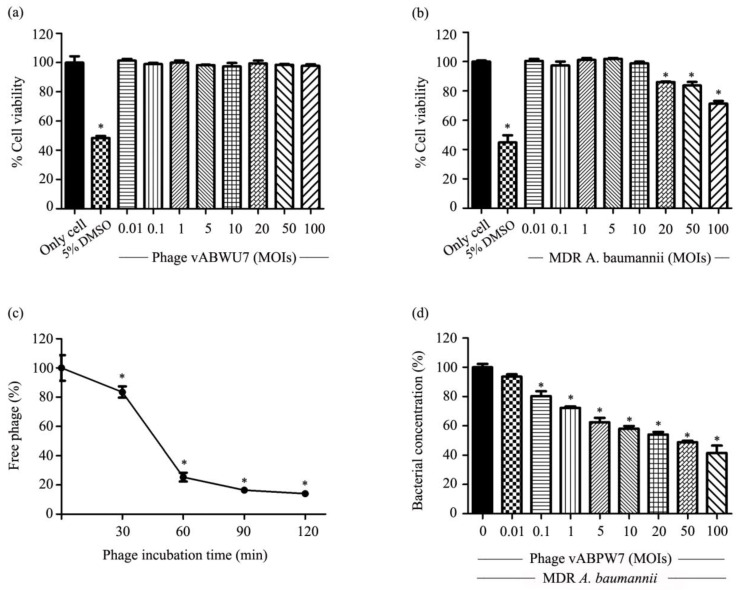
Efficacy of phage vABPW7 in A549 cell culture model. Cytotoxicity of phage vABWU7 towards A549 cells at MOIs of 0.01 to 100 (**a**) and MDR *A. baumannii* at MOIs of 0.01 to 100 (**b**) after incubation for 24 h. (**c**) Adsorption efficacy of phage vABPW7 to A549 cells. (**d**) Efficacy of phage vABWU7 at MOIs of 0.01 to 100 to reduce MDR *A. baumannii* under the A549 cell culture model. Experiments were undertaken independently in duplicate with the duplicate assay. The data show the mean ± SEM (* *p* value < 0.05).

The cytotoxicity of phage and MDR *A. baumannii* on A549 cells was evaluated using the MTT assay at 24 h post-treatment. Phage vABPW7 at MOIs of 0.01 to 100 did not induce cytotoxicity when compared with a control group—cells treated with a combination of DMEM and SM buffer (Figure 7a). For the cytotoxicity effects of MDR *A. baumannii* on the cells, there was no significant difference between cell viability of MDR *A. baumannii*-infected cells at MOIs of 0.01 to 10 and the control group—cells treated with DMEM treatment (Figure 7b). A significant induction of cytotoxicity was observed after MDR *A. baumannii* treatment at MOIs of 20, 50, and 100.

### 3.13. Adsorption of Phage to Human Lung Epithelial Cells

The adsorption efficacy of phage vABPW7 into A549 cells was assessed every 30 min for 2 h post-incubation. The results (Figure 7c) showed that about 75% of the phage were adsorbed to A549 cells within 1 h, and by 2 h post-incubation, approximately 85% of phage vABPW7 was adsorbed to the cells.

### 3.14. Screening Effect of Phage vABPW7 on MDR A. baumannii-Infected A549 Cells

The effect of phage vABPW7 on MDR *A. baumannii*-infected A549 cells was determined by a resazurin fluorometric assay; in addition, cell viability was assessed in parallel by an MTT cell viability assay. A549 cells were infected with MDR *A. baumannii* at an MOI of 1 and then treated with phage vABPW7 at MOIs of 0.01 to 100, in parallel with a control group—A549 cells infected with MDR *A. baumannii*. The results showed that a reduction in the total fluorescence was detected after treatment with phage vABPW7 at an MOI of 0.01 (Figure 7d). The phage at MOIs of 0.1 to 100 significantly reduced the percentage of total fluorescence compared to the control. There were no significant differences in cell viability in each condition when assessed by the MTT assay (Supplementary Figure S1a).

### 3.15. Kinetic Assay of the Phage on Planktonic Bacteria

**Figure 8:**
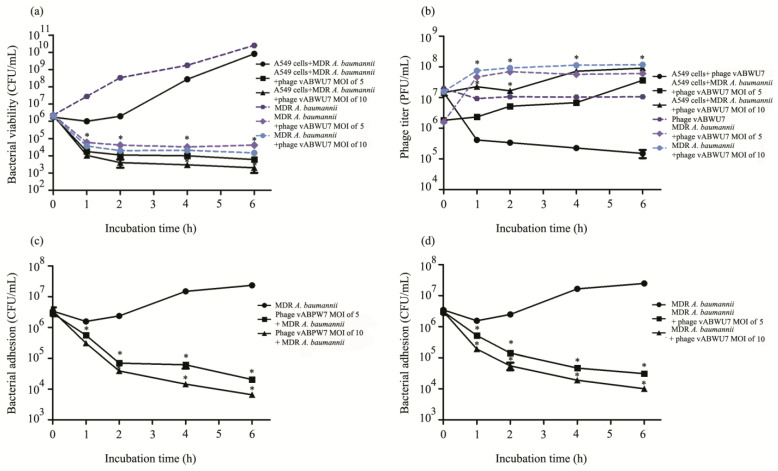
Efficacy of phage vABPW7 to reduce the number of planktonic and adhered MDR *A. baumannii* on A549 cells. Kinetics of the antibacterial activity of phage vABWU7 on planktonic bacteria under the cell culture model (**a**) was determined in parallel with the production of phage vABWU7 (**b**). The ability of phage vABWU7 to reduce bacterial attachment to the surface of A549 cells was investigated. For a prophylactic model, A549 cells were treated with phage vABWU7 and then infected with MDR *A. baumannii.* The number of adherent bacteria was assessed at the times indicated (**c**). For a therapeutic model, A549 cells were infected with MDR *A. baumannii* and then treated with phage vABWU7. The number of adherent bacteria was assessed at the times indicated (**d**).

A kinetic assay of the phage on planktonic MDR *A. baumannii* in the A549 cell culture model was evaluated and compared to a model without A549 cells. A549 cells were infected with MDR *A. baumannii* and then treated with phage vABPW7. The supernatant was collected at 0, 1, 2, 4, and 6 h post-incubation followed by the quantification of planktonic bacteria cell viability and phage vABPW7. At 6 h post-incubation, the viability of cells was determined by the trypan blue exclusion test. In the A549 cell culture model, the number of planktonic MDR *A. baumannii* in the cells infected with the bacteria condition was reduced at 1 h. The number of planktonic MDR *A. baumannii* gradually increased after 2 h post-incubation. For the phage treatment group, the phage at MOIs of 5 and 10 significantly reduced planktonic MDR *A. baumannii* at 1 h post-incubation, and the planktonic bacteria was still reduced at 6 h post-incubation (Figure 8a). The number of phage vABPW7 in the supernatant was also measured in parallel. The results of the phage-treated cell condition showed the free phage titer decreasing over the first 1 h post-incubation (Figure 8b). For the phage treatment on bacterially infected cells, the phage titer at 2 h was significantly increased, and at 6 h post-incubation, the phage was produced in both models; the phage at an MOI of 5 and the phage at an MOI of 10 was significantly higher than that in control—cells incubated with only phage vABPW7 at an MOI of 10.

In the model without A549 cells, the number of MDR *A. baumannii* in a control group without the phage treatment increased continuously (Figure 8a). Treatment with phage vABPW7 at MOIs of 5 and 10 reduced the number of MDR *A. baumannii* at 1 h post-infection over the 6 h period. The phage titer was also determined. The phage titer under the conditions—the phage at an MOI of 5 infected MDR *A. baumannii* and the phage at an MOI of 10 infected MDR *A. baumannii*—was gradually induced at 1 h post-infection (Figure 8b). At 6 h post-incubation, the titer of phage under the condition of MDR *A. baumannii* infected with phage at an MOI of 10, was significantly higher than the condition of phage at an MOI of 5 infected MDR *A. baumannii.* The phage titer under both conditions, phage at an MOI of 5 treated MDR *A. baumannii*-infected cells and phage at an MOI of 10 treated MDR *A. baumannii*-infected cells, was significantly higher than the control—phage alone. In addition, there were no cytotoxicity effects on A549 cells when assessed by a trypan blue exclusion test (Supplementary Figure S1b).

### 3.16. Efficacy of Phage to Reduce Bacterial Attachment

To evaluate the phage efficacy in reducing bacterial adhesion into A549 cells, prophylactic and therapeutic treatment models were performed. Attached bacterial cell viability and phage production under each parameter were determined at 0, 1, 2, 4, and 6 h post-incubation by counting the bacterial colony and plaque assay, respectively. Moreover, the A549 cell viability was also determined in parallel at 6 h post-incubation by a trypan blue exclusion test. The cells were incubated with phage vABPW7 and then infected with MDR *A. baumannii* in the prophylactic model. The results (Figure 8c) showed that phage vABPW7 at MOIs of 5 and 10 significantly decreased bacterial adhesion into A549 cells at various time points. At 6 h post-incubation, the adhered MDR *A. baumannii* was reduced by approximately 3.3 log and 3.6 log after phage treatment at MOIs of 5 and 10, respectively. The results indicated a dose-dependent reduction in the number of adhered MDR *A. baumannii* cells. No cytotoxic effects were detected in each condition (Supplementary Figure S1c).

In the therapeutic model, MDR *A. baumannii* was added to the cells which were then treated with phage vABPW7, and the efficacy of phage vABPW7 in decreasing bacterial attachment into A549 cells was determined. The degree of MDR *A. baumannii* attachment was reduced by approximately 2.9 log and 3.5 log after the treatment of phage vABPW7 at MOIs of 5 and 10 compared with the untreated control (Figure 8d). The results indicated that phage vABPW7 reduced the attached bacteria in a dose-dependent manner. No cytotoxicity was detected when compared with the control (Supplementary Figure S1d). There was no significant difference in bacterial cell viability between the efficacy of prophylactic and therapeutic treatments.

### 3.17. Ultrastructure of Bacterial Cells in the Adhesion Assay

**Figure 9:**
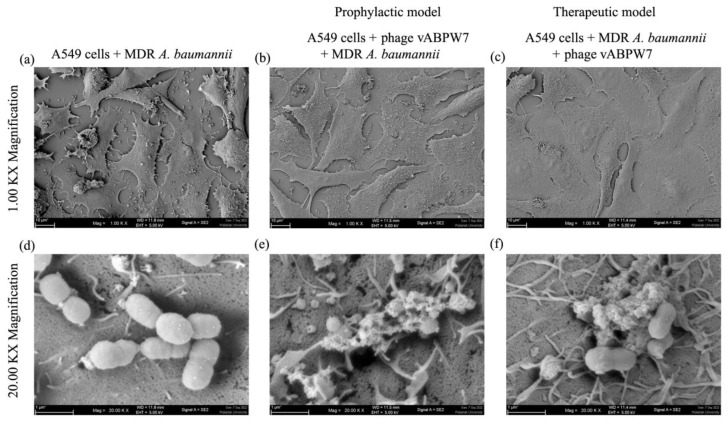
Ultrastructural analysis of MDR *A. baumannii* that attached to the cell surface of A549 cells under FE-SEM. (**a**,**d**) A549 cells incubated with only MDR *A. baumannii*. (**b**,**e**) A549 cells were treated with phage vABWU7 and then infected with MDR *A. baumannii* as a prophylactic application. (**c**,**f**) A549 cells were infected with MDR *A. baumannii* and then treated with phage vABWU7 as a therapeutic application. The cells were observed at magnifications of ×1000 and ×20,000.

The effect of phage vABPW7 on MDR *A. baumannii* cell attached to A549 cells was confirmed under FE-SEM. A549 cells were incubated with phage vABPW7 followed by MDR *A. baumannii* infection as a prophylactic treatment, while A549 cells were infected with MDR *A. baumannii* followed by the phage treatment as a therapeutic treatment. The number of MDR *A. baumannii* on A549 cells after the phage treatment were reduced when compared with a control of only bacteria-infected cells (Figure 9a–c). MDR *A. baumannii* was a coccobacillus bacteria with an intact cell membrane (diameter 0.57 ± 0.16 μm and length 0.88 ± 0.15 μm (n = 3)) (Figure 9d). Phage vABPW7 induced morphological changes in the MDR *A. baumannii* cell. Wrinkled surfaces, membrane damage, and pore formation were observed, leading to bacterial cell death (diameter 0.6 ± 0.05 μm and length 1.43 ± 0.12 μm (n = 3)) (Figure 9e,f). Some leaked contents and debris were detected around bacterial cells. There were no differences in the bacterial structural changes between prophylactic and therapeutic treatments. In addition, a trypan blue exclusion test was performed in parallel to confirm cell viability (Supplementary Figure S1e). The phage, MDR *A. baumannii*, and both phage and MDR *A. baumannii* treatments did not have cytotoxic effects on the viability of A549 cells, indicating that phage vABPW7 was safe and possible to use in phage therapy.

## 4. Discussion

MDR *A. baumannii* is an emerging pathogen associated with increased morbidity and mortality rates worldwide. MDR *A. baumannii* is frequently involved in hospital-acquired and ventilator-associated pneumonia. However, MDR *A. baumannii* has become a common pathogen, which can infect the respiratory tract, blood, soft tissues, and urinary tract, resulting in septicemia, meningitis, endocarditis, pneumonia, wound, and urinary tract infections. MDR *A. baumannii* rapidly develops resistance to all antibiotics, including colistin and tigecycline—the only effective antibiotics in clinical treatment [47]. Given the severity of the current situation, there is considerable global interest in developing alternative treatment options for infections caused by MDR *A. baumannii* strains including the development of new antibiotics, nanoparticles, peptides, and phages. To date, various phages infecting *A. baumannii* have been studied and reported, and *Acinetobacter* phages have been investigated in vitro and in vivo, including their use in clinical trials in the US [19,48]. However, no report has been published on the efficacy of the *Acinetobacter* phage in a cell culture model. To further shed some light on phage therapy, this study focused on the isolation and characterization of a phage infecting MDR *A. baumannii*, determined the effects of this phage on biofilm, and evaluated the efficacy of phage in vitro, including in an A549 cell culture model.

Phage vABPW7, which has a long and contractile tail, was enriched and isolated from a wastewater sample. The phage produces small plaques on an MDR *A. baumannii* lawn, and the plaques have a halo around them indicating that the phage might encode depolymerases with polysaccharide-degrading activity. A phage host range and EOP analysis showed that phage vABPW7 was able to infect 45% of 20 clinical strains. These results indicated that phage vABPW7 could possibly be used in phage therapy, especially as part of a phage cocktail. An increase in the number of bacterial strains for host range testing might provide useful additional information. Many common factors affecting phage viability, such as temperature, pH, UV radiation, and phage infectivity over long periods, have been reported [49]. Phage vABPW7 could tolerate a broad range of exposure to acidic and base conditions, different temperatures, glycerol storage, and UV radiation, suggesting that it might be possible to further develop this phage in pharmaceutical formulations such as lyophilized and freeze-dried phages. To infect bacterial cells, phages interact and then enter the cells. Phage vABPW7 has a short latency period and a large burst size that produces a large number of progeny phage. The in vitro biological assays showed that even when a low MOI of phage was used, phage vABPW7 still reduced the turbidity of MDR *A. baumannii* completely, highlighting the high lytic activity of phage vABPW7.

The genome study of phage vABPW7 showed that the phage is classified as a member of the genus *Phapecoctavirus*, subfamily *Stephanstirmvirinae*, class *Caudoviricetes,* and order *Caudovirales*. The comparative whole-genome sequencing results showed no *Acinetobacter* phage-sharing whole-genome similarity with phage vABPW7. Interestingly, phage vABPW7 shares the highest whole-genome identity with *Escherichia* phage BI-EHEC. The phage also shares high sequence similarity with *Klebsiella* phage ZCKP1, Dompiswa phage TSP7_1, and *Myoviridae* sp. Even though most of the studies on phages have reported that phages are highly host-specific, some phages can infect many species and different genera of bacteria [50]. Thus, it is possible that phage vABPW7, which has similar genome sequences to *Escherichia* phage, could infect *A. baumannii.* The identity of phage vABPW7 and *Escherichia* phage BI-EHEC was 95.1%, indicating that phage vABPW7 might be a typical phage in the genus *Phapecoctavirus*, subfamily *Stephanstirmvirinae*, class *Caudoviricetes*, and order *Caudovirales*. Furthermore, there was no virulence factor or resistance gene in the genome of phage vABPW7, suggesting the potential as a reliable therapeutic agent for phage therapy against MDR *A. baumannii*. Due to the high similarity of phage vABPW7 and *Escherichia* phage-specific avian pathogenic *E. coli*, the lytic activity of phage vABPW7 towards Avian pathogenic *E. coli* should be investigated in the future.

The process of a phage entering bacterial cells is mediated by a specific interaction between the bacterial cell membrane and the tail fiber protein, which is a phage receptor-binding protein functioning in phage adsorption and penetration into bacterial cells [51]. The binding of bacteria and phage results in the opening of a central channel of the tail required for DNA release from the capsid. Normally, tail fiber proteins will bind to a specific host protein, determining the specificity of a host–phage interaction [52]. The genetic relationship of phage vABPW7 was analyzed, and the tail fiber protein was selected as a model based on its specificity. The tail fiber protein of phage vABPW7 shared some sequence similarities to the tail fiber protein of *Escherichia* phage vB B_EcoM_ESCO25 and *Escherichia* phage ESCO13, demonstrating that phage vABPW7 was grouped into the genus *Phapecoctavirus*, corresponding to the whole-genome analysis. In addition, previous studies have shown that the tail fiber proteins of phage, such as *Acinetobacter* phage IME200 [53], *Acinetobacter* phage phiAB6 [54], and *Klebsiella* phage SH-KP152226 [55], possess depolymerase activity which can degrade polysaccharides such as capsular polysaccharides and extracellular polysaccharides, removing a physical barrier to allow access to the bacterial host cell receptors [56]. Capsular polysaccharides and extracellular polysaccharides are the major structural components of biofilms, which are communities of bacterial cells. Biofilm, one crucial virulence factor, is challenging to control as it limits antibiotic penetration and aids in the evasion of the host immune system [57]. It is well documented that a halo around a plaque usually indicates depolymerase activity of a phage [58], and as discussed above, a small halo around the plaques produced by phage vABPW7 was observed. The anti-biofilm activity of phage vABPW7 was investigated, and the phage possesses good efficacy in reducing biofilm formation and removing preformed biofilms, consistent with previous reports showing that phage kills bacterial cells, leading to the prevention of biofilm formation [59]. Moreover, phages can penetrate preformed biofilm and then remove the biofilm structure. Our results suggest that phage vABPW7 might be suitable to develop as a biocontrol agent aimed at preventing and removing biofilm formation. Further characterization of phage-derived enzymes for biofilm control should be carried out.

MDR *A. baumannii* can cause various diseases, including pneumonia, an infection that inflames the alveoli in the lung. Cases of nosocomial pneumonia, severe community-acquired pneumonia, and ventilator-associated pneumonia have been reported to continuously increase [60]. Previous studies have reported that *A. baumannii* can adhere, colonize, and invade human epithelial cells, followed by intracellular multiplication, dissemination to other tissues, persistence, and activation of cell death pathways [61]. Human lung epithelial cells (A549 cells) are frequently used as a model to investigate *A. baumannii* infections in mammalian cells. *A. baumannii* interacts with A549 cells through fibronectin and then enters the cells, leading to pathogenesis [62,63]. Recently, the mechanism of phage entry into mammalian cells was elucidated—phages were transported across epithelial cell layers and the phages were internalized to cells via macropinocytosis [64]. In addition, the adsorption of phage to various mammalian cells was reported and A549 lung epithelial cells showed the highest accumulation of phage [65]. To ensure confidence in the safety of phage vABPW7, the cytotoxicity of phage vABPW7 towards A549 cells was investigated. The results showed no cytotoxicity towards A549 cells after phage vABPW7 treatment, indicating the safety of phage vABPW7. These results corresponded with previous reports that phages are not toxic to cells and phage-mediated bacterial lysis also did not induce the cytotoxicity [20]. Moreover, interactions of phage and epithelial cells does not cause cell injury [65]. In an adhesion experiment, this study showed that phage vABPW7 could reduce bacterial adhesion. When the attached bacteria are reduced, the reduced adherent bacteria might result in a reduction in biofilm formation, reduced bacterial invasion, and reduced intracellular bacteria. The results showed that planktonic bacteria levels were also reduced, suggesting that phage vABPW7 might kill free-living planktonic cells before the planktonic cells attach to the host cells. Applying phage as a bacterial anti-adhesive agent to surfaces might help decrease biofilm formation, bacterial attachment, and bacterial contamination. Previous studies have shown that the presence of phages affects the bacterial and mammalian host interface, and that interactions between phages and bacteria reduces microbial colonization and pathology [66]. However, the influence of phage inside cells remains largely unknown [64], and the mechanism of the antibacterial activity of phage in a mammalian cell model warrants further investigation.

## 5. Conclusions

*Acinetobacter* phage vABPW7 has the potential to be used as an alternative antibacterial approach to control MDR *A. baumannii* infections. Phage vABPW7 is suitable for further development towards its application in phage therapy, including as part of a phage cocktail.

## Figures and Tables

**Table 1:** Host range infection and EOP of phage vABPW7.

Strain	Phage vABPW7	
	Lytic Activity	EOP
MDR *A. baumannii* ABPW060	+	Low (0.04)
MDR *A. baumannii* ABPW061	-	-
MDR *A. baumannii* ABPW062	-	-
MDR *A. baumannii* ABPW063	+	High (Host = 1)
MDR *A. baumannii* ABPW064	-	-
MDR *A. baumannii* ABPW065	-	-
MDR *A. baumannii* ABPW066	+	Low (0.01)
MDR *A. baumannii* ABPW067	-	-
MDR *A. baumannii* ABPW068	-	-
MDR *A. baumannii* ABPW069	-	-
MDR *A. baumannii* ABPW070	+	Moderate (0.26)
MDR *A. baumannii* ABPW071	+	High (0.61)
MDR *A. baumannii* ABPW072	-	-
MDR *A. baumannii* ABPW073	+	Moderate (0.1)
MDR *A. baumannii* ABPW074	-	-
MDR *A. baumannii* ABPW075	+	Moderate (0.4)
MDR *A. baumannii* ABPW076	-	-
MDR *A. baumannii* ABPW077	+	High (0.67)
MDR *A. baumannii* ABPW078	+	Moderate (0.38)
MDR *A. baumannii* ABPW079	-	-
*A*. *baumannii* ATCC 17978	-	-
*E. coli* ECPW01	-	-
*K. pneumoniae* KPPW01	-	-
MRSA PW01	-	-
*P. aeruginosa* PAPW01	-	-

+ Able to produce lytic zone, - was unable to produce lytic zone. The spot-test-positive strains were selected to perform the EOP test. The EOP values were calculated by the ratio of phage titer on the test bacterium relative to the phage titer on the host bacteria. High production efficiency is EOP  ≥  0.5, moderate production efficiency is 0.5  >  EOP  ≥  0.1, low production efficiency is 0.1  >  EOP  >  0.001, and the inefficiency of phage production is EOP ≤ 0.001. Experiments were undertaken independently in duplicate with the duplicate assay.

## Data Availability

Not applicable.

## Supplementary Materials

The following supporting information can be downloaded at: https://www.mdpi.com/article/10.3390/v14112561/s1, Figure S1: Cytotoxicity in A549 cells.; Table S1: Drug resistance profiles of MDR A. *baumannii.*; Table S2: Annotation of the specific function of ORFs in phage vABWU7 genome was conducted using Blastx.; Table S3: Annotation of the specific function of ORFs in phage vABWU7 genome was conducted using EggNOG.

## References

[1] Upmanyu K., Haq Q.M.R., Singh R. (2022). Factors mediating Acinetobacter baumannii biofilm formation: Opportunities for developing therapeutics. Curr. Res. Microb. Sci..

[2] Beachey E.H. (1981). Bacterial adherence: Adhesin-receptor interactions mediating the attachment of bacteria to mucosal surface. J. Infect. Dis..

[3] Choi C.H., Lee J.S., Lee Y.C., Park T.I., Lee J.C. (2008). Acinetobacter baumannii invades epithelial cells and outer membrane protein A mediates interactions with epithelial cells. BMC Microbiol..

[4] Brossard K.A., Campagnari A.A. (2012). The Acinetobacter baumannii biofilm-associated protein plays a role in adherence to human epithelial cells. Infect. Immun..

[5] Singh R., Capalash N., Sharma P. (2022). Vaccine development to control the rising scourge of antibiotic-resistant Acinetobacter baumannii: A systematic review. 3 Biotech.

[6] Principi N., Silvestri E., Esposito S. (2019). Advantages and Limitations of Bacteriophages for the Treatment of Bacterial Infections. Front. Pharmacol..

[7] Sharma S., Chatterjee S., Datta S., Prasad R., Dubey D., Prasad R.K., Vairale M.G. (2017). Bacteriophages and its applications: An overview. Folia Microbiol..

[8] Cruz-Lopez F., Martinez-Melendez A., Villarreal-Trevino L., Morfin-Otero R., Maldonado-Garza H., Garza-Gonzalez E. (2022). Contamination of healthcare environment by carbapenem-resistant Acinetobacter baumannii. Am. J. Med. Sci..

[9] Koskella B., Meaden S. (2013). Understanding bacteriophage specificity in natural microbial communities. Viruses.

[10] Sisakhtpour B., Mirzaei A., Karbasizadeh V., Hosseini N., Shabani M., Moghim S. (2022). The characteristic and potential therapeutic effect of isolated multidrug-resistant Acinetobacter baumannii lytic phage. Ann. Clin. Microbiol. Antimicrob..

[11] Yele A.B., Thawal N.D., Sahu P.K., Chopade B.A. (2012). Novel lytic bacteriophage AB7-IBB1 of Acinetobacter baumannii: Isolation, characterization and its effect on biofilm. Arch. Virol..

[12] Wintachai P., Surachat K., Singkhamanan K. (2022). Isolation and Characterization of a Novel Autographiviridae Phage and Its Combined Effect with Tigecycline in Controlling Multidrug-Resistant Acinetobacter baumannii-Associated Skin and Soft Tissue Infections. Viruses.

[13] Wintachai P., Voravuthikunchai S.P. (2022). Characterization of Novel Lytic Myoviridae Phage Infecting Multidrug-Resistant Acinetobacter baumannii and Synergistic Antimicrobial Efficacy between Phage and Sacha Inchi Oil. Pharmaceuticals.

[14] Udekwu K.I., Parrish N., Ankomah P., Baquero F., Levin B.R. (2009). Functional relationship between bacterial cell density and the efficacy of antibiotics. J. Antimicrob. Chemother..

[15] Grygorcewicz B., Wojciuk B., Roszak M., Lubowska N., Blazejczak P., Jursa-Kulesza J., Rakoczy R., Masiuk H., Dolegowska B. (2021). Environmental Phage-Based Cocktail and Antibiotic Combination Effects on Acinetobacter baumannii Biofilm in a Human Urine Model. Microb. Drug Resist..

[16] Sausset R., Petit M.A., Gaboriau-Routhiau V., De Paepe M. (2020). New insights into intestinal phages. Mucosal Immunol..

[17] Jeon J., Park J.H., Yong D. (2019). Efficacy of bacteriophage treatment against carbapenem-resistant Acinetobacter baumannii in Galleria mellonella larvae and a mouse model of acute pneumonia. BMC Microbiol..

[18] Wintachai P., Naknaen A., Thammaphet J., Pomwised R., Phaonakrop N., Roytrakul S., Smith D.R. (2020). Characterization of extended-spectrum-beta-lactamase producing Klebsiella pneumoniae phage KP1801 and evaluation of therapeutic efficacy in vitro and in vivo. Sci. Rep..

[19] Schooley R.T., Biswas B., Gill J.J., Hernandez-Morales A., Lancaster J., Lessor L., Barr J.J., Reed S.L., Rohwer F., Benler S. (2017). Development and Use of Personalized Bacteriophage-Based Therapeutic Cocktails To Treat a Patient with a Disseminated Resistant Acinetobacter baumannii Infection. Antimicrob. Agents Chemother..

[20] Shan J., Ramachandran A., Thanki A.M., Vukusic F.B.I., Barylski J., Clokie M.R.J. (2018). Bacteriophages are more virulent to bacteria with human cells than they are in bacterial culture; insights from HT-29 cells. Sci. Rep..

[21] Moller-Olsen C., Ho S.F.S., Shukla R.D., Feher T., Sagona A.P. (2018). Engineered K1F bacteriophages kill intracellular Escherichia coli K1 in human epithelial cells. Sci. Rep..

[22] Porayath C., Salim A., Palillam Veedu A., Babu P., Nair B., Madhavan A., Pal S. (2018). Characterization of the bacteriophages binding to human matrix molecules. Int. J. Biol. Macromol..

[23] Bonilla N., Rojas M.I., Netto Flores Cruz G., Hung S.H., Rohwer F., Barr J.J. (2016). Phage on tap-a quick and efficient protocol for the preparation of bacteriophage laboratory stocks. PeerJ.

[24] Ackermann H.W. (2009). Phage classification and characterization. Methods Mol. Biol..

[25] Kutter E. (2009). Phage host range and efficiency of plating. Methods Mol. Biol..

[26] Kropinski A.M. (2009). Measurement of the rate of attachment of bacteriophage to cells. Methods Mol. Biol..

[27] Montso P.K., Mlambo V., Ateba C.N. (2019). Characterization of Lytic Bacteriophages Infecting Multidrug-Resistant Shiga Toxigenic Atypical Escherichia coli O177 Strains Isolated From Cattle Feces. Front. Public Health.

[28] Chandrarathna H., Nikapitiya C., Dananjaya S.H.S., De Silva B.C.J., Heo G.J., De Zoysa M., Lee J. (2020). Isolation and characterization of phage AHP-1 and its combined effect with chloramphenicol to control Aeromonas hydrophila. Braz. J. Microbiol..

[29] Wintachai P., Phaonakrop N., Roytrakul S., Naknaen A., Pomwised R., Voravuthikunchai S.P., Surachat K., Smith D.R. (2022). Enhanced antibacterial effect of a novel Friunavirus phage vWU2001 in combination with colistin against carbapenem-resistant Acinetobacter baumannii. Sci. Rep..

[30] Niu Y.D., Liu H., Johnson R.P., McAllister T.A., Stanford K. (2020). Effect of a bacteriophage T5virus on growth of Shiga toxigenic Escherichia coli and Salmonella strains in individual and mixed cultures. Virol. J..

[31] Meier-Kolthoff J.P., Goker M., Sproer C., Klenk H.P. (2013). When should a DDH experiment be mandatory in microbial taxonomy?. Arch. Microbiol..

[32] Meier-Kolthoff J.P., Auch A.F., Klenk H.P., Goker M. (2013). Genome sequence-based species delimitation with confidence intervals and improved distance functions. BMC Bioinform..

[33] Meier-Kolthoff J.P., Goker M. (2017). VICTOR: Genome-based phylogeny and classification of prokaryotic viruses. Bioinformatics.

[34] Lefort V., Desper R., Gascuel O. (2015). FastME 2.0: A Comprehensive, Accurate, and Fast Distance-Based Phylogeny Inference Program. Mol. Biol. Evol..

[35] Drummond A.J., Ho S.Y., Phillips M.J., Rambaut A. (2006). Relaxed phylogenetics and dating with confidence. PLoS Biol..

[36] Goker M., Garcia-Blazquez G., Voglmayr H., Telleria M.T., Martin M.P. (2009). Molecular taxonomy of phytopathogenic fungi: A case study in Peronospora. PLoS ONE.

[37] Meier-Kolthoff J.P., Klenk H.P., Goker M. (2014). Taxonomic use of DNA G+C content and DNA-DNA hybridization in the genomic age. Int. J. Syst. Evol. Microbiol..

[38] Nishimura Y., Yoshida T., Kuronishi M., Uehara H., Ogata H., Goto S. (2017). ViPTree: The viral proteomic tree server. Bioinformatics.

[39] Altschul S.F., Madden T.L., Schaffer A.A., Zhang J., Zhang Z., Miller W., Lipman D.J. (1997). Gapped BLAST and PSI-BLAST: A new generation of protein database search programs. Nucleic Acids Res..

[40] Lee H.W., Koh Y.M., Kim J., Lee J.C., Lee Y.C., Seol S.Y., Cho D.T., Kim J. (2008). Capacity of multidrug-resistant clinical isolates of Acinetobacter baumannii to form biofilm and adhere to epithelial cell surfaces. Clin. Microbiol. Infect..

[41] Srisuwan S., Voravuthikunchai S.P. (2017). Rhodomyrtus tomentosa Leaf Extract Inhibits Methicillin-Resistant Staphylococcus aureus Adhesion, Invasion, and Intracellular Survival in Human HaCaT Keratinocytes. Microb. Drug Resist..

[42] Wintachai P., Paosen S., Yupanqui C.T., Voravuthikunchai S.P. (2019). Silver nanoparticles synthesized with Eucalyptus critriodora ethanol leaf extract stimulate antibacterial activity against clinically multidrug-resistant Acinetobacter baumannii isolated from pneumonia patients. Microb. Pathog..

[43] Oelschlaeger T.A., Tall B.D. (1997). Invasion of cultured human epithelial cells by Klebsiella pneumoniae isolated from the urinary tract. Infect. Immun..

[44] Magiorakos A.P., Srinivasan A., Carey R.B., Carmeli Y., Falagas M.E., Giske C.G., Harbarth S., Hindler J.F., Kahlmeter G., Olsson-Liljequist B. (2012). Multidrug-resistant, extensively drug-resistant and pandrug-resistant bacteria: An international expert proposal for interim standard definitions for acquired resistance. Clin. Microbiol. Infect..

[45] Kropinski A.M., Prangishvili D., Lavigne R. (2009). Position paper: The creation of a rational scheme for the nomenclature of viruses of Bacteria and Archaea. Environ. Microbiol..

[46] Rice P., Longden I., Bleasby A. (2000). EMBOSS: The European Molecular Biology Open Software Suite. Trends Genet..

[47] Vazquez-Lopez R., Solano-Galvez S.G., Juarez Vignon-Whaley J.J., Abello Vaamonde J.A., Padro Alonzo L.A., Rivera Resendiz A., Muleiro Alvarez M., Vega Lopez E.N., Franyuti-Kelly G., Alvarez-Hernandez D.A. (2020). Acinetobacter baumannii Resistance: A Real Challenge for Clinicians. Antibiotics.

[48] LaVergne S., Hamilton T., Biswas B., Kumaraswamy M., Schooley R.T., Wooten D. (2018). Phage Therapy for a Multidrug-Resistant Acinetobacter baumannii Craniectomy Site Infection. Open Forum Infect. Dis..

[49] Duyvejonck H., Merabishvili M., Vaneechoutte M., de Soir S., Wright R., Friman V.P., Verbeken G., De Vos D., Pirnay J.P., Van Mechelen E. (2021). Evaluation of the Stability of Bacteriophages in Different Solutions Suitable for the Production of Magistral Preparations in Belgium. Viruses.

[50] Ross A., Ward S., Hyman P. (2016). More Is Better: Selecting for Broad Host Range Bacteriophages. Front. Microbiol..

[51] Scholl D., Rogers S., Adhya S., Merril C.R. (2001). Bacteriophage K1-5 encodes two different tail fiber proteins, allowing it to infect and replicate on both K1 and K5 strains of Escherichia coli. J. Virol..

[52] North O.I., Davidson A.R. (2021). Phage Proteins Required for Tail Fiber Assembly Also Bind Specifically to the Surface of Host Bacterial Strains. J. Bacteriol..

[53] Liu Y., Mi Z., Mi L., Huang Y., Li P., Liu H., Yuan X., Niu W., Jiang N., Bai C. (2019). Identification and characterization of capsule depolymerase Dpo48 from Acinetobacter baumannii phage IME200. PeerJ.

[54] Lai M.J., Chang K.C., Huang S.W., Luo C.H., Chiou P.Y., Wu C.C., Lin N.T. (2016). The Tail Associated Protein of Acinetobacter baumannii Phage PhiAB6 Is the Host Specificity Determinant Possessing Exopolysaccharide Depolymerase Activity. PLoS ONE.

[55] Wu Y., Wang R., Xu M., Liu Y., Zhu X., Qiu J., Liu Q., He P., Li Q. (2019). A Novel Polysaccharide Depolymerase Encoded by the Phage SH-KP152226 Confers Specific Activity Against Multidrug-Resistant Klebsiella pneumoniae via Biofilm Degradation. Front. Microbiol..

[56] Drulis-Kawa Z., Majkowska-Skrobek G., Maciejewska B. (2015). Bacteriophages and phage-derived proteins--application approaches. Curr. Med. Chem..

[57] Anderl J.N., Franklin M.J., Stewart P.S. (2000). Role of antibiotic penetration limitation in Klebsiella pneumoniae biofilm resistance to ampicillin and ciprofloxacin. Antimicrob. Agents Chemother..

[58] Fernandes S., Sao-Jose C. (2018). Enzymes and Mechanisms Employed by Tailed Bacteriophages to Breach the Bacterial Cell Barriers. Viruses.

[59] Chang C., Yu X., Guo W., Guo C., Guo X., Li Q., Zhu Y. (2022). Bacteriophage-Mediated Control of Biofilm: A Promising New Dawn for the Future. Front. Microbiol..

[60] Xu A., Zhu H., Gao B., Weng H., Ding Z., Li M., Weng X., He G. (2020). Diagnosis of severe community-acquired pneumonia caused by Acinetobacter baumannii through next-generation sequencing: A case report. BMC Infect. Dis..

[61] Pizarro-Cerda J., Cossart P. (2006). Bacterial adhesion and entry into host cells. Cell.

[62] Smani Y., McConnell M.J., Pachon J. (2012). Role of fibronectin in the adhesion of Acinetobacter baumannii to host cells. PLoS ONE.

[63] Gaddy J.A., Tomaras A.P., Actis L.A. (2009). The Acinetobacter baumannii 19606 OmpA protein plays a role in biofilm formation on abiotic surfaces and in the interaction of this pathogen with eukaryotic cells. Infect. Immun..

[64] Bichet M.C., Chin W.H., Richards W., Lin Y.W., Avellaneda-Franco L., Hernandez C.A., Oddo A., Chernyavskiy O., Hilsenstein V., Neild A. (2021). Bacteriophage uptake by mammalian cell layers represents a potential sink that may impact phage therapy. iScience.

[65] Gorski A., Borysowski J., Miedzybrodzki R. (2020). Bacteriophage Interactions With Epithelial Cells: Therapeutic Implications. Front. Microbiol..

[66] Barr J.J., Auro R., Furlan M., Whiteson K.L., Erb M.L., Pogliano J., Stotland A., Wolkowicz R., Cutting A.S., Doran K.S. (2013). Bacteriophage adhering to mucus provide a non-host-derived immunity. Proc. Natl. Acad. Sci. USA.

