# Supplementary figures and images for: Isolation and Characterization of a Phapecoctavirus Infecting Multidrug-Resistant Acinetobacter baumannii in A549 Alveolar Epithelial Cells
Source: Viruses. 2022 Nov 19;14(11):2561. doi: 10.3390/v14112561 (PMC9695679; doi:10.3390/v14112561)

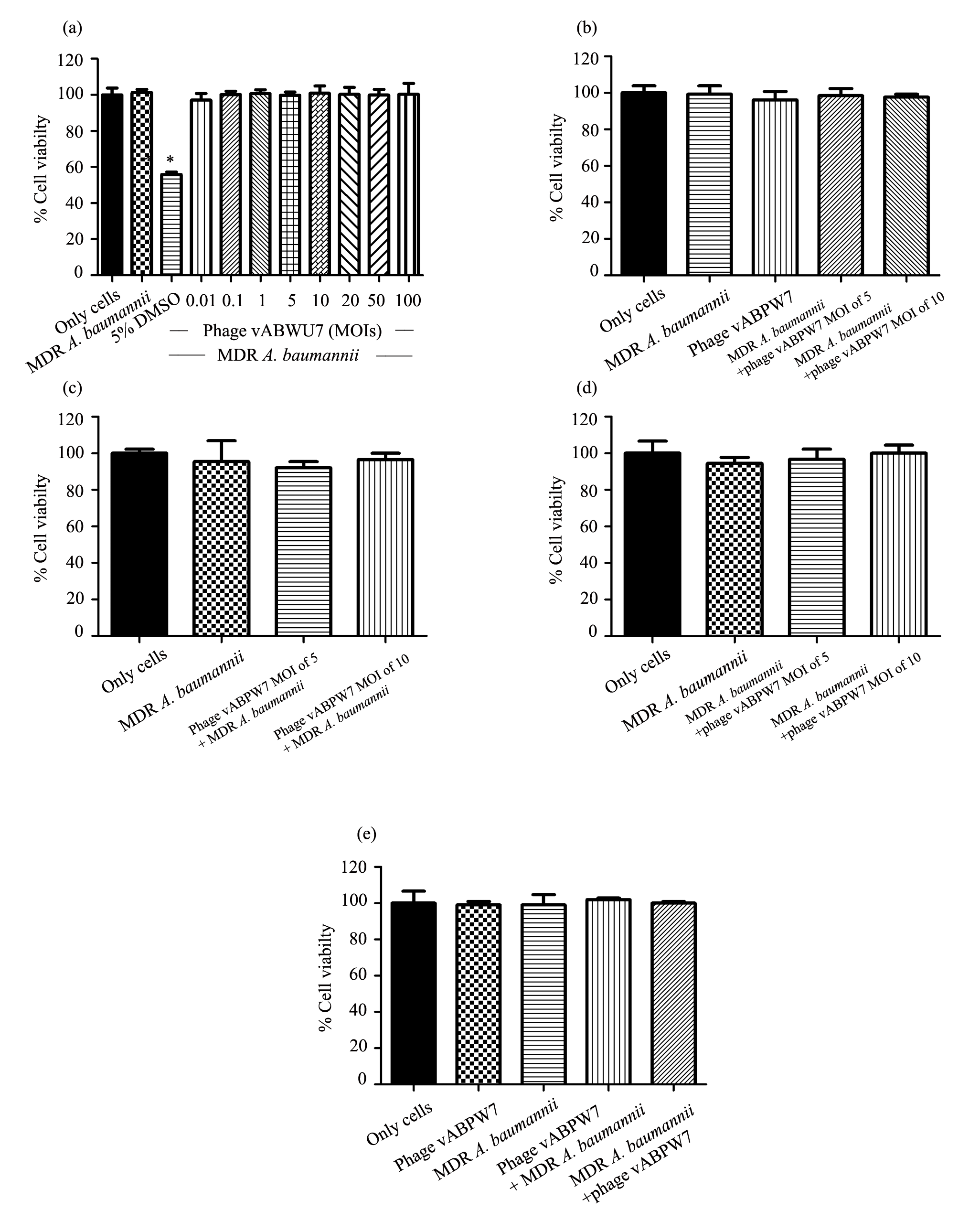

Supplement: Supplementary file 1 [file viruses-14-02561-s001.zip › Supplementary Figure S1.tif]
